# Reproxalap in patients with seasonal allergic conjunctivitis: a systematic review and meta-analysis

**DOI:** 10.1186/s12348-025-00497-3

**Published:** 2025-04-28

**Authors:** Ammar Elgadi, Malaz M. Abdalmotalib, Tibyan Noorallah, Egbal Abdelazim, Fatma Ali Merghani Mohammed

**Affiliations:** Faculty of Medicine, University of Khartoum, Khartoum, Sudan

**Keywords:** Seasonal allergic conjunctivitis, Reactive aldehyde species, Reproxalap, Ocular itching, Meta-analysis

## Abstract

**Introduction:**

Seasonal allergic conjunctivitis (SAC) is a hypersensitivity condition characterized by itching, tearing, and redness. It affects over 20% of the general population with limited therapeutic options. Reproxalap, a novel small-molecule aldehyde trap, has emerged as a potential treatment option for SAC by targeting reactive aldehydes involved in inflammation. In this systematic review and meta-analysis, we assessed the efficacy and safety of Reproxalap in treating SAC.

**Methods:**

Multiple databases were searched including PubMed, Cochrane Library, Scopus, and Google Scholar, to identify relevant studies. Clinical trials involving patients diagnosed with SAC and treated with Reproxalap (0.25% or 0.5%) were included. The primary outcomes were symptom relief (efficacy) and side effects (safety). Data extraction and risk of bias assessment were performed independently by two reviewers. Meta-analysis was conducted using RevMan 5.4 software.

**Results:**

Five RCTs involving 625 participants were included. Reproxalap significantly reduced ocular itching compared to control groups for both 0.25% (SMD: -0.31, 95% CI: -0.50 to -0.13, *P* = .001) and 0.5% concentrations (SMD: -0.31, 95% CI: -0.51 to -0.10, *P* = 0.004). No significant difference was observed between the two doses (SMD: -0.02, 95% CI: -0.23 to 0.19, *P* = 0.83). Side effects were more frequent in both Reproxalap groups compared to controls, with odds ratios of 35.31 (95% CI: 17.83 to 69.90, *P* < 0.00001) for 0.25% and 45.64 (95% CI: 18.49 to 112.66, *P* < 0.00001) for 0.5%. The 0.5% dose had significantly higher odds of side effects compared to the 0.25% dose (OR: 1.66, 95% CI: 1.10 to 2.51, *P* = 0.02). Heterogeneity was low for all outcomes (I^2^ = 0%).

**Conclusion:**

Reproxalap reduces ocular itching associated with SAC. While both 0.25% and 0.5% concentrations are effective, safe and tolerable. Further research should focus on the long-term benefits and applicability of Reproxalap on diverse populations.

**Supplementary Information:**

The online version contains supplementary material available at 10.1186/s12348-025-00497-3.

## Summary

- Reproxalap significantly reduced ocular itching in SAC patients compared to controls.
- Both 0.25% and 0.5% doses showed similar efficacy.
- Side effects were higher in Reproxalap groups, with more in the 0.5% dose but no added benefit.
- No serious adverse events were reported, indicating a generally safe profile.

## Introduction

Seasonal allergic conjunctivitis (SAC) is an inflammatory response of the conjunctiva triggered by exposure to seasonal allergies [1]. It is an IgE-mediated, hypersensitivity condition characterized by itching, tearing, and redness [2].

SAC affects over 20% of the global population, making it one of the most prevalent ocular allergic conditions worldwide and a significant contributor to the global health burden [3, 4]. Beyond its high prevalence, SAC poses substantial socioeconomic and healthcare challenges. Symptoms such as itching, redness, tearing, and photophobia can severely impair daily activities, including reading, driving, and using digital devices, leading to reduced productivity and quality of life. Additionally, the condition is associated with increased absenteeism from work or school and a growing economic burden due to frequent healthcare visits and ongoing medication costs [5].

Despite this widespread impact, the development of novel therapeutic options for SAC has remained stagnant for decades, leaving patients with limited options for effective long-term management.

Current first-line treatments, including topical antihistamines and mast cell stabilizers, provide symptom relief for many patients. However, these therapies often fall short in managing moderate to severe cases, with up to 60% of patients requiring additional interventions to control persistent or severe symptoms [6, 7]. For these individuals, topical corticosteroids are frequently used as second-line therapy. While corticosteroids effectively reduce inflammation and alleviate symptoms, their prolonged use is associated with significant risks, including elevated intraocular pressure, glaucoma, cataract formation, and other ocular complications. These risks limit their suitability for long-term management and highlight a critical unmet need for safer, more effective therapies [8, 9].

The ongoing burden of SAC underscores the urgency of advancing research and clinical strategies to develop more targeted and sustainable treatment options. Reproxalap is a novel chemical entity undergoing late-stage clinical development for ocular inflammation. It targets reactive aldehyde species (RASP), a specific class of small molecules. Reproxalap modulates protein structure and function by sequestering RASP through covalent binding to amine and thiol residues [10, 11]. This interaction alters RASP activity, which is typically upregulated during inflammation and acts before cytokine release, activating critical inflammatory mediators such as nuclear factor kappa B and inflammasomes [12, 13]. This mechanism has potential applications across various inflammatory ocular conditions, including allergic conjunctivitis, noninfectious anterior uveitis, and dry eye disease [14–16].

In this review, we aimed to consolidate evidence on reproxalap’s efficacy and safety from various clinical trials. Therefore, providing a comprehensive assessment of its therapeutic potential.

## Methods

The protocol for this systematic review was conducted according to the Preferred Reporting Items for Systematic Reviews and Meta-Analyses (PRISMA) (Supplementary 1).

### Information sources

The databases PubMed, Cochrane Library, Scopus, and Google Scholar were searched for the eligible articles.

### Search strategy

We developed an inclusive search strategy. Our full search strategy was “Reproxalap OR (2-(3-amino- 6-chloroquinolin- 2-yl)propan- 2-ol) OR (ALD- 102) OR (ADX- 102)”. The search strategy for each database was provided in (supplementary 2).

### Eligibility criteria

Articles were eligible if they were reports of randomized controlled trials (RCTs) with 2 or more arms, investigating patients diagnosed with seasonal allergic conjunctivitis (SAC), treated with Reproxalap regardless of the dose compared to vehicle. Articles not reporting outcomes of interest, namely efficacy (symptom relief) or safety (side effects), were excluded. There were no restrictions on geographical location or healthcare setting.

### Study selection

**Fig. 1:**
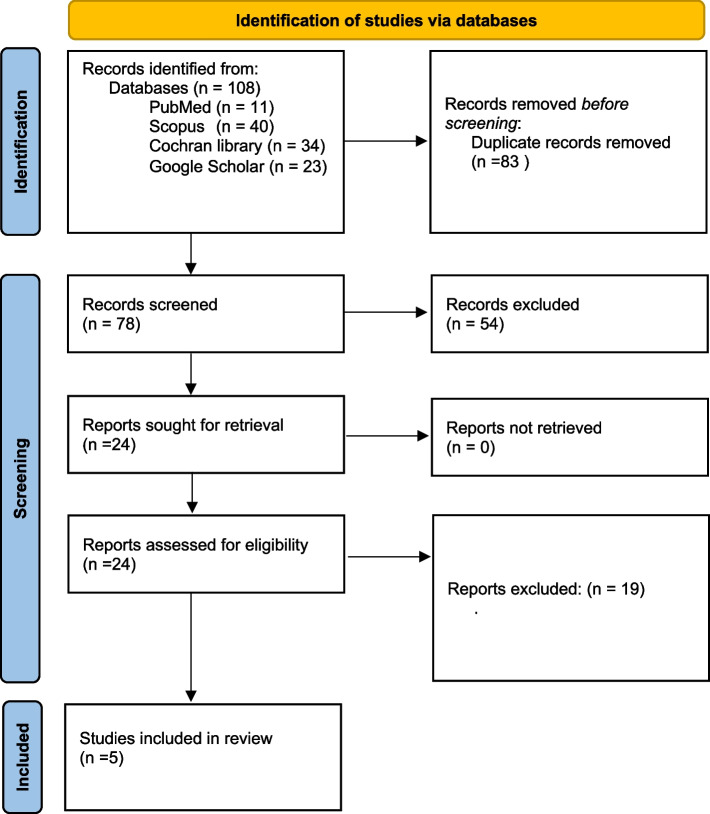
PRISMA flow diagram showing the study selection process. From: Page MJ, McKenzie JE, Bossuyt PM, Boutron I, Hoffmann TC, Mulrow CD, et al. The PRISMA 2020 statement: an updated guideline for reporting systematic reviews. BMJ 2021;372:n71. https://doi.org/10.1136/bmj.n71

The database records were uploaded to EndNote reference management software to remove duplicates. The duplicates were removed based on the author's name and DOI similarity. Then A screening was initiated using a Microsoft Excel spreadsheet. One independent reviewer screened the titles and abstracts for relevance. Then there was the full-text screening for eligibility. Another reviewer revised all the screening phases for validity. When there were discrepancies, they were solved by discussion between the two reviewers (Fig. 1).

### Data collection process

Data extraction was done by two independent authors one used a pre-designed spreadsheet and the other used Google NotebookLM. Then the data was compared revised and edited manually by the two authors. The extracted data include the study design, number of arms, and intervention details. Also, we extracted the baseline characteristics of the participants. Lastly, we extracted data on outcomes of interest, including ocular tearing score, ocular itching score, ocular redness score, eyelid swelling, and side effects including treatment-emergent adverse events (TEAEs). Two reviewers independently extracted the data and cross-verified it for accuracy.

### Risk of bias assessment

The risk of bias assessment was done using the Cochrane Risk of Bias tool for RCTs [17]. We evaluated five key domains including randomization process, deviations from intended interventions, missing outcome data, measurement of outcomes, and selection of reported results. Each domain was rated as low risk, some concerns, or high risk of bias.

### Data synthesis

A descriptive narrative synthesis was provided for the baseline characteristics and the outcomes that were ineligible for meta-analysis. Meta-analysis was conducted using RevMan 5.4. A fixed effect model was used as the default. The random effect model was used when there was high heterogeneity. We used the mean and standardized mean differences as pooled effect size estimates. However, the standard deviation in the study by Clark et al. [18] was not reported, so we used the standard deviation from their previous 2021 study. All the effect size measures were reported with 95% confidence intervals. Heterogeneity was assessed using the I^2^ statistic. Cavanagh et al. [15] report the mean difference in the efficacy outcomes, though this is more statistically sound, the other studies did not report sufficient data to calculate the mean difference. In our meta, a combination of mean difference and mean was inappropriate; hence, we excluded Cavanagh et al. [] from the meta-analysis of the efficacy outcomes.

## Results

### Characteristics of included studies and participants demographics

Our review included a total of 625 participants from five randomized clinical trials. The majority of the participants were female. Regarding ethnicity, the majority were non-Hispanic and non-Latino followed by white, black, or African American, and a smaller representation of Asian participants. Brown eyes were the most frequent eye color, followed by black and blue eyes. Gray eyes were the least common among the participants (Table 1).

**Table 1:** Summary of the included studies'characteristics and participants'demographics

Study ID	Cavanagh et al.—2022 [15]	Starr et al.—2023 [10]	Clark et al.—2021 [19]	Clark et al.—2022 [18]	NCT03012165
Design	RCT phase 2	RCT phase 3 (crossover)	RCT phase 3	RCT phase 2 (crossover)	RCT phase 2
arms	0.25 0.5 Vehicle	0.5 Vehicle	0.25 0.5 Vehicle	0.25 0.5 Vehicle	0.1 0.5 Vehicle
Total Number	52	89	318	66	154
Age (Mean, SD)	45.2 (14.27)	41.1 (12.25)	45.8 (14.47)	46.9 (10.07)	-
Females	35	42	197	-	97
Hispanic or Latino	30	0	46	9	12
Not Hispanic or Latino	22	-	272	61	141
Asian	1	27	35	9	2
Black or African American	2	19	47	14	48
White	48	55	232	45	68
Other Race	1	4	4	2	36
Black Iris	5	-	78	-	68
Blue Iris	8	0	78	-	29
Brown Iris	33	0	173	-	86
Hazel Iris	4	-	33	-	18
Green Iris	5	-	29	-	19
Gray Iris	0	-	3	-	1

*RCT* Randomized clinical trial, *SD* Standard deviation

### Risk of bias assessment

**Fig. 2:**
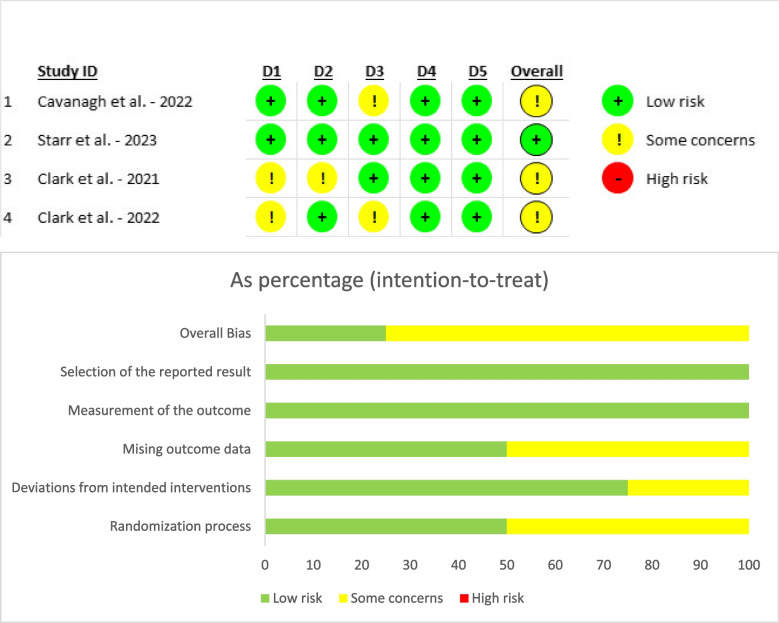
Risk of bias assessment using ROB2. * D1: Randomisation process, D2: Deviations from the intended interventions, D3: Missing outcome data, D4: Measurement of the outcome, D5: Selection of the reported result

Assessment of bias of the included studies reveals overall some concerns to low risk. The outcome measurement domain was consistently rated as low risk across all sources. Cavanagh et al. [15] exhibited low risk in randomization, intervention deviations, outcome measurement, and selective reporting, though risks were unclear due to missing data. Similarly, Clark et al. [19] showed low risk in outcome data, outcome measurement, and reporting, with some concerns about randomization and intervention deviations. In Clark et al. [18], intervention deviations, outcome measurement, and result selection posed low risks, yet randomization and missing data remained unclear. Starr et al. [10] demonstrated low risk in all domains (Fig. 2).

### Efficacy

- Ocular itching score

**Fig. 3:**
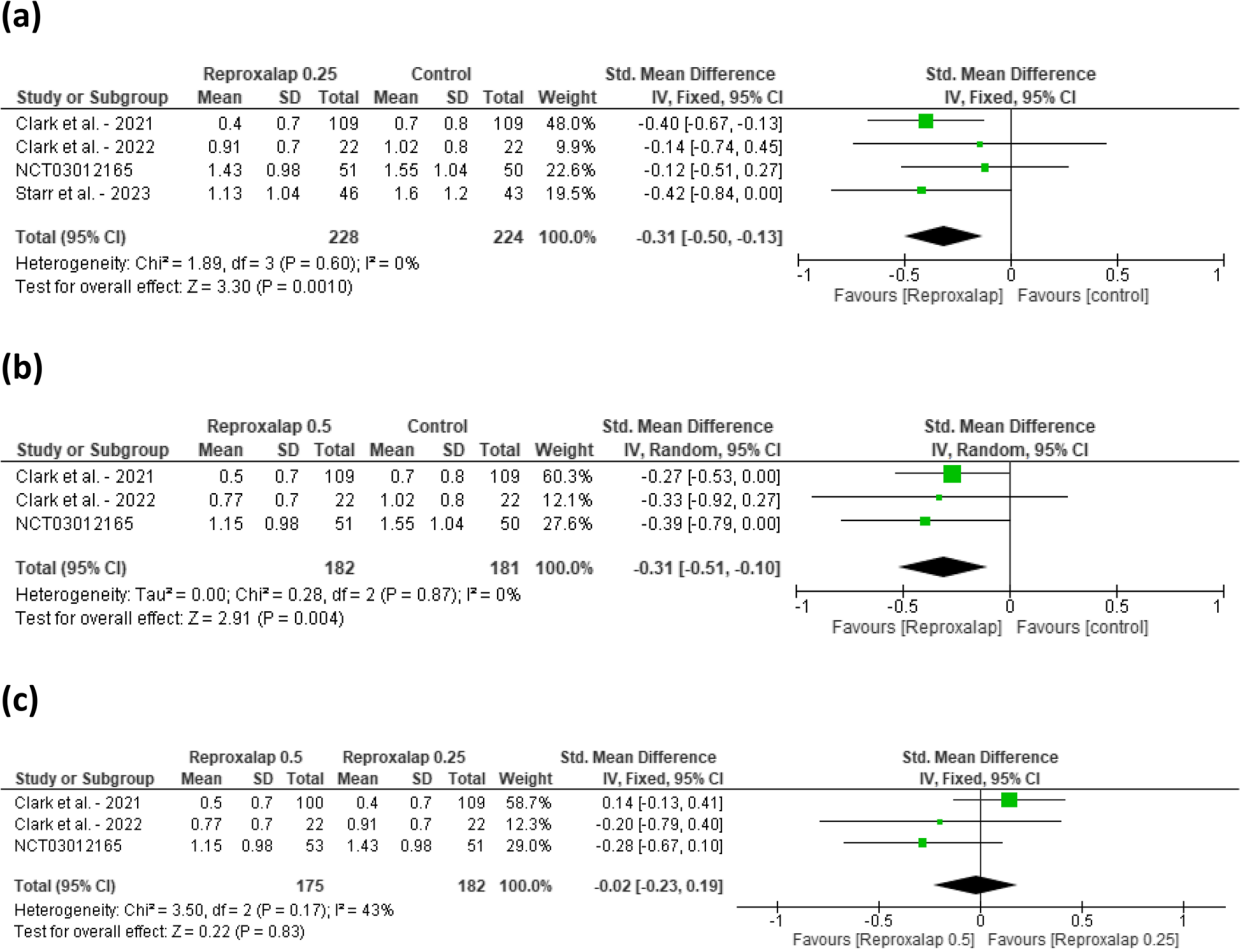
Forest plot comparing the effect of reproxalap 0.25% and 0.5% on ocular itching scores against control. **a** Reproxalap 0.25% vs. control. **b** Reproxalap 0.5% vs. control (**c**) Reproxalap 0.25% vs. 0.5%

Comparing Reproxalap 0.25 against control the pooled standard mean difference (SMD) for the ocular itching score was − 0.31 [95% CI: − 0.50, − 0.13] favoring Reproxalap 0.25%. The test for the overall effect was statistically significant (*P* = 0.0010). Heterogeneity among the studies was minimal (I^2^ = 0%) (Fig. 3a). For Reproxalap 0.5, the pooled standard mean difference (SMD) for the ocular itching score was − 0.31 [95% CI: − 0.51, − 0.10] favoring Reproxalap 0.5%. The test for the overall effect was statistically significant (*P* = 0.004). Heterogeneity among the studies was minimal (I^2^ = 0%) (Fig. 3b). Comparing the two doses, the standard mean difference (SMD) for the ocular itching score was − 0.02 [95% CI: − 0.23, 0.19]. The test for the overall effect was not statistically significant (*P* = 0.83). Heterogeneity among the studies was minimal (I^2^ = 0%) (Fig. 3c).

- Ocular Tearing score

In Cavanagh et al. [15], the mean change in ocular tearing was − 0.11 ± 0.04 for the 0.25% group, − 0.32 ± 0.07 for the 0.50% group, and 0.36 ± 0.11 for the control group. Starr et al. [10] reported a mean of 0.63 ± 0.77 for the 0.25% group, with no data provided for the 0.50% and control groups. Clark et al. [19] found a mean of 0.62 ± 0.77 for the 0.25% group, 0.51 ± 0.77 for the 0.50% group, and 0.73 ± 0.85 for the control group.

- Ocular Redness score

In Cavanagh et al. [15], the mean change in ocular redness was − 0.59 (0.09) for the 0.25% group, − 0.42 ± 0.09 for the 0.50% group, and − 0.045 ± 0.17 for the control group. Starr et al.[10] observed a mean of 1.6 ± 1.2 for the 0.25% group, with no data available for the 0.50% group. Clark et al. [19] reported a mean of 0.73 ± 0.8 for the 0.25% group, 0.79 for the 0.50% group, and 0.85 for the control group. In study NCT03012165, the mean was 1.43 ± 0.98 for the treatment group and 1.55 ± 1.04 for the control.

- Eyelid Swelling

Cavanagh et al. [15] reported mean changes in eyelid swelling of − 0.35 (0.06) for the 0.25% group, − 0.53 ± 0.12 for the 0.50% group, and 0.024 ± 0.09 for the control group. Starr et al.[10] observed a change of 0.59 ± 0.56 for the 0.25% group, with no data for the 0.50% group.

### Side effects

**Fig. 4:**
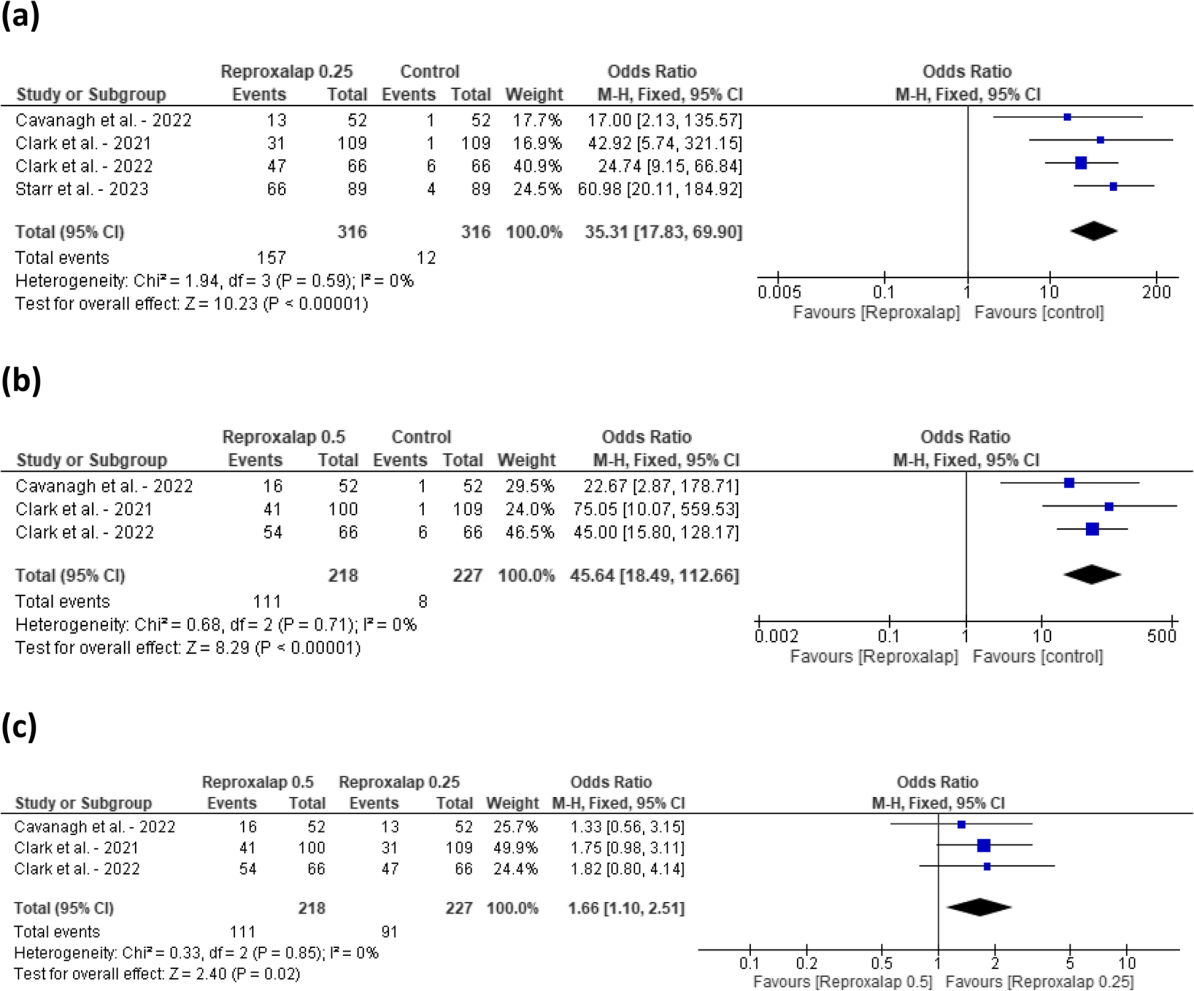
Forest plot comparing the effect of reproxalap 0.25% and 0.5% on ocular side effects against control. **a** Reproxalap 0.25% vs. control. **b** Reproxalap 0.5% vs. control (**c**) Reproxalap 0.25% vs. 0.5%

Comparing Reproxalap 0.25 against control, the pooled odds ratio (OR) for side effects was 35.31 [95% CI: 17.83, 69.90]. The test for overall effect was significant (*P* < 0.00001). This indicates a higher incidence of side effects in the Reproxalap 0.25% group compared to the control group. Heterogeneity among the studies was low (I^2^ = 0%) (Fig. 4a). While for Reproxalap 0.5, the pooled odds ratio (OR) for side effects was 45.64 [95% CI: 18.49, 112.66]. The test for overall effect was significant (*P* < 0.00001). This indicates a higher incidence of side effects in the Reproxalap 0.5% group compared to the control group. Heterogeneity among the studies was low (I^2^ = 0%) (Fig. 4b). Comparing the two doses, the pooled odds ratio (OR) for side effects was 1.66 [95% CI: 1.10, 2.51]. The test for the overall effect was significant (*P* = 0.02). Heterogeneity among the studies was low (I^2^ = 0%) (Fig. 4c).

## Discussion

Our results demonstrated the efficacy and safety of Reproxalap in relieving symptoms of SAC like itching, redness, and swelling of eyelids in a cohort of 625 participants. Our analysis showed that both 0.25% and 0.5% Reproxalap concentrations significantly reduced ocular itching compared to control groups. However, no significant difference was found between the two concentrations and the 0.5% dose was not adding incremental benefit. Similar to itching, ocular redness, tearing, and eyelid swelling were reduced in the Reporxalap groups compared to vehicles. This reduction of symptoms was true in both cases when Repoxalap was used to prevent the occurrence of symptoms or when used at the peak of symptoms which reflects prophylactic as well as treatment activity as stated by Clark et al. [18].

Reproxalap offers a more targeted strategy for reducing ocular inflammation by selectively targeting reactive aldehyde species (RASP) [20]. RASPs are integral to initiating and maintaining inflammation, particularly in the post-histaminic phase of allergic reactions. They contribute to ongoing cellular infiltration and other sub-acute physiological changes in allergic conjunctivitis [21, 22]. Hence, unlike antihistamines, which block histamine receptors to address the early phase of allergic reactions, Reproxalap works downstream in the inflammatory process [20, 22]. Neutralizing RASP helps to modulate a network of proteins involved in the inflammatory cascade, thus providing relief from both acute and ongoing symptoms [15]. Compared to corticosteroids, which broadly suppress inflammation but carry significant side effects, Reproxalap offers a safer, more targeted alternative [20]. This mechanism makes it particularly valuable for patients with more persistent or severe symptoms.

Traditional treatments for SAC each exhibit distinct limitations, Antihistamines while providing prompt relief for itching, primarily address the early phase of allergic reactions and are less effective in managing the subsequent inflammation. Mast cell stabilizers, though beneficial in preventing histamine release, require prolonged use to achieve efficacy, making them suboptimal for acute symptom relief. Corticosteroids are potent anti-inflammatory agents Capable of controlling severe symptoms; however, their long-term use is associated with significant risks, including cataracts, glaucoma, and elevated intraocular pressure, particularly if it is used for prolonged periods [9, 23, 24]. Nonsteroidal anti-inflammatory drugs (NSAIDs), though generally safer, offer weaker anti-inflammatory effects and have the potential risk of inducing corneal irritation, which limits their use in severe cases [25, 26].

Reproxalap offers a promising therapeutic option by addressing the limitations of existing treatments. It combines the rapid symptom relief typically provided by antihistamines with the potent anti-inflammatory effects of corticosteroids but without the associated ocular and systemic adverse effects. Through its targeted action on reactive aldehyde species (RASP), Reproxalap modulates the inflammatory cascade at multiple stages, effectively managing both the early and late phases of allergic response in SAC.

Regarding the safety of Reproxalap, both concentrations were associated with a higher incidence of side effects compared to control groups; however, no severe adverse events were observed, and no patients had to discontinue treatment due to side effects.

In any of the included studies. The most common side effect of Reproxalap was mild and transient irritation at the site of installation. This was reported in 23–78% of patients using Reproxalap, compared to just 2–9% of those receiving the vehicle. this irritation was generally mild and resolved on its own, it was slightly more common with the 0.5% dose.

Our findings are consistent with those reported by Mandell et al., 2020. They found that transient mild ocular discomfort was the most frequently observed side effect in patients using Reproxalap for non-infectious anterior uveitis, and this did not affect overall treatment tolerability [27]. In addition to their findings, we may argue that this irritation is unlikely to stop most patients from continuing therapy, especially when they are informed about it beforehand. Clinicians should take the opportunity to explain that this side effect is both temporary and harmless, which can help to maintain adherence. The lack of significant findings in visual acuity, intraocular pressure, and other ocular health parameters further supports Reproxalap's safety.

The comparable efficacy of 0.25% and 0.5% concentrations highlights the potential superiority of using the lower dose in clinical practice since it minimizes side effects while preserving therapeutic values. Reproxalap's safety profile has been consistently demonstrated across various conditions, including noninfectious anterior uveitis [27] and dry eye disease [28]. In SAC patients the most commonly reported side effect was mild and transient instillation site irritation [19], indicating that Reproxalap is generally well tolerated. Given the similar efficacy observed between higher and lower concentrations, prioritizing the lower dose may offer an optimal balance between safety and efficacy. However, further studies comparing different dosages are essential to establish the most effective and safe concentration for broader clinical applications.

Our study had important limitations to declare. First, is the lack of important numerical values for the meta-analysis. This was solved using a mathematical approach as discussed in the method section, however, this approach may reflect only an approximation of the true values founded by the original studies. Second, the number of trials was only five with different durations of treatment and variations in the outcomes reporting which made conducting more analysis much more difficult. Third, the trials included in this analysis enrolled white participants predominantly raising concerns about the generalizability of the findings. Given that allergic conjunctivitis may present differently across ethnic groups, and that genetic variations can affect treatment responses, future research should include a more diverse participant pool to enhance the applicability of Reproxalap across broader populations. Finally, there is a lack of data on long-term outcomes, patient-reported satisfaction, and quality-of-life measures, all of which are critical for assessing the overall benefits. These gaps should be addressed through comprehensive, long-term studies.

## Conclusion

Reproxalap was effective in relieving symptoms of allergic conjunctivitis, particularly itching, redness, and swelling. Both low and high doses appear effective and safe; however, careful attention to dosing is needed. Further research should focus on the long-term benefits and applicability of Reproxalap on diverse populations.

### Artificial intelligence AI use

The authors declare using AI (NotebookLM, Consensus, Quilbot) for data extraction, finding relevant articles, and language editing and paraphrasing. All the information was reviewed manually and edited by the authors. Interest

## Supplementary Information

Supplementary Material 1.

Supplementary Material 2.

## Data Availability

Data supporting the findings of this study are available within the manuscript.
