# Supplementary material for: Reproxalap in patients with seasonal allergic conjunctivitis: a systematic review and meta-analysis
Source: J Ophthalmic Inflamm Infect. 2025 Apr 28;15:39. doi: 10.1186/s12348-025-00497-3 (PMC12037956; doi:10.1186/s12348-025-00497-3)
Supplement: Supplementary file 2 — Supplementary Material 2. [file 12348_2025_497_MOESM2_ESM.docx]

**Full search strategy**

| **Data base** | **Search strategy** | **Number of records found** | **Date of search** |
| --- | --- | --- | --- |
| **PubMed** | Reproxalap OR (2-(3-amino-6-chloroquinolin-2-yl)propan-2-ol) OR (ALD-102) OR (ADX-102) | **11** | **7/19/2024** |
| **Scopus** | Reproxalap OR (ALD-102) OR (ADX-102) | **40** | **7/19/2024** |
| **Cochrane library** | Reproxalap OR (2-(3-amino-6-chloroquinolin-2-yl)propan-2-ol) OR (ALD-102) OR (ADX-102) | **34** | **7/19/2024** |
| **Google Scholar** | Reproxalap OR (ALD-102) OR (ADX-102) | **23** | **7/19/2024** |
